# Prediction of Heterosis for Agronomic Traits in Half-Diallel Cross of Rice (*Oryza sativa* L.) under Drought Stress Using Microsatellite Markers

**DOI:** 10.3390/plants11121532

**Published:** 2022-06-08

**Authors:** Khaled F. M. Salem, Mousa A. Alghuthaymi, Abdelmoaty B. Elabd, Elsayed A. Elabsawy, Hossam H. Mierah

**Affiliations:** 1 Plant Biotechnology Department, Genetic Engineering and Biotechnology Research Institute (GEBRI), University of Sadat City, Sadat City 22857, Egypt; salemcairo@yahoo.com; 2 Biology Department, Science and Humanities College, Shaqra University, Alquwayiyah 11726, Saudi Arabia; 3 Field Crops Research Institute, Agricultural Research Center (ARC), Giza 33717, Egypt; abdelmoaty@rocketmail.com; 4 Bioinformatics Department, Genetic Engineering and Biotechnology Research Institute (GEBRI), University of Sadat City, Sadat City 22857, Egypt; khaled.salem@gebri.usc.edu.eg

**Keywords:** agronomic traits, drought, F_1_-hybrids, heterosis, genetic distance, rice (*Oryza sativa* L.)

## Abstract

Rice breeders are encouraged to classify potential F_1_-hybrids without crossing all viable mixtures by testing depending on genetic distance due to lack of labor and time in yield trials. The goals of this research were to establish heterosis and investigate the relationship between genomic distance and agronomic attributes under drought. Half-diallel mating design, 28 F_1′_^s^ and 8 parents were evaluated under drought and genotyped using 11 microsatellite markers. In total, 39 alleles were detected. Results indicated that the greatest heterotic effects for grain yield were observed in Sakha 103 × Sakha 104 and GZ7576-10-3-2-1 × Giza 179, which gave 29.32–22.57% heterosis, respectively. Heterosis for grain yield in these crosses occurred as a rise in panicle weight, filled grains per panicle, low sterility and 100-grain weight. Correlations of marker-based genetic distance with mid-parent heterosis were positively and significantly correlated with sterility percentage (*r* = 0.390 *, *p* < 0.05). However, better-parent heterosis was positively and significantly correlated with sterility percentage (*r* = 0.352 *, *p* < 0.05) and grain yield per plant (*r* = 0.345 *, *p* < 0.05). Associations indicate that high grain yield and low sterility of rice crosses can be expected from microsatellite marker-defined distances of parents. This study indicated that genetic distance is very effective for heterosis prediction in breeding programs.

## 1. Introduction

Rice (*Oryza sativa* L.) is the greatest essential nutrition crop in the globe since it is the staple food for nearly 50% of the world population [1,2]. Around 490.27 million metric tons of rice were consumed universally in the 2018/2019 agricultural season, while 437.18 million metric tons were consumed in the 2008/2009 crop season [1]. In 2019, the world’s top 10 main rice-producing nations were China, India, Indonesia, Bangladesh, Vietnam, Thailand, Myanmar, Philippines, Japan and Brazil [1]. In Egypt, during the 2019 rice-growing season, the area cultivated with rice was 485,622.54 hectares with an average of 8.37 t/h and total production was 4.8 million tons [1]. Major developments have happened in rice production because of the broad-scale adoption of developed rice varieties. However, the request for rice in low-income nations remains to be increased because of increases in public rice customers and developments in living standards. It is expected that the globe will need to yield 50% additional rice by 2050. To meet this task, high-yielding potential genotypes are needed. Several approaches have been employed for developing new genotypes with superior yielding potential and abiotic stress tolerance, such as population development, ideotype breeding, heterosis, wide crossing, genetic engineering and molecular breeding [3,4,5]. Rice is considered the greatest common and essential field crop in Egypt for numerous reasons as a (i) essential food after wheat for the Egyptian population, (ii) second exporting crop after cotton and (iii) soil reclamation crop for developing the production of the salty soil crops in which all farmers could increase money during its growing season. Rice productivity and production have remarkably improved year after year. Because of adopting the new short-duration rice genotypes, about 30% of the irrigation water use was saved each year [6].

Drought is severe abiotic stress that can cause serious losses of yield and productivity in most crop plants in dry and semi-dry regions [7]. Egypt is self-sufficient in rice, but due to a high population growth rate, the ongoing process of climatic changes and the decline of Egypt’s share of the river Nile water, rice production may decline to levels insufficient to maintain self-sufficiency. The development of water stress-tolerant genotypes that maintain excellent yield under drought is a priority area of rice study for sustainable rice production [7].

Hybrid improvement is an expensive and time-consuming procedure. Identifying the parental combinations that produce superior offspring is critical, and one of the extra expensive aspects of the development of excellent hybrids. Previous studies that were carried out on significant heterosis were reported for plant height and grain yield/plant [8]. Other traits, namely panicle length, number of panicles/plant, sterility% and 100-grain weight, were examined [9]. In self-pollinated crops such as rice, the better abrades are to develop a true-breeding homogeneous population with superior agronomic and other desirable traits. The accomplishment of these objectives would depend on the suitable choices of the parental material, nature of gene action controlling characters under consideration and rational choice of breeding method for bringing out quick and maximum genetic improvement. This would imply the basic knowledge of the genetic behavior of the characters under improvement, which is a pre-request for the breeder to manipulate the breeding material to isolate superior lines [10,11].

Diallel analysis has been used in recent years by many breeders to evaluate parental materials before taking any decision regarding the style of breeding system to be used in this concern [10]. Knowledge of the genetic diversity and associations between rice genotypes is necessary for identifying promising combinations for the exploitation of heterosis and the establishment of heterotic collections for use as source resources in rice breeding programs [12,13,14,15]. Increasing evidence indicates that there is a strong relationship between the genetic distance among parents and F_1_ performance or heterosis for some traits [16,17,18]. Coefficients of correlation among microsatellite-based genetic distance and heterosis mainly for grain yield have ranged from not significant to significantly strong. However, there is still a lack of knowledge about the association between genetic distance and cross success in rice germplasm; additionally, the factors that contribute to low correlations must be clarified.

The aims of the present study are to (i) assess the heterosis of 28 hybrids under drought stress, (ii) investigate the relationship among microsatellite-based genetic distance and heterosis effects of agronomic trait and yield efficiency and (iii) identify the best cross combinations that can be used for further objectives in rice breeding programs. These research goals will provide reliable information that will aid rice breeders in detecting promising genotypes and verifying whether hybrid prediction based on microsatellite genetic distance is sufficient.

## 2. Results

### 2.1. Informativeness of Microsatellite Markers

In total, 11 microsatellite markers were tested for their ability to generate microsatellite banding patterns from DNA corresponding and evaluate the genetic diversity of 8 Egyptian and exotic rice varieties. All microsatellite markers utilized in the current investigation produced polymorphic fragments, producing a polymorphism rate of 100% among the 6 varieties from different origins (Table 1). A total of 39 alleles were identified. The alleles number per locus varied from 2 for locus RM 162 with a size range of 67–295 bp to 4 for RM 1, RM 19, RM 144, RM 271, RM 316, RM 338, RM 452 with a mean number of 3.54 alleles per locus (Table 1).

### 2.2. Gene Diversity

Polymorphic information content (PIC), a measure of gene diversity, for the 11 microsatellites ranged from 0.456 for RM 338 to 0.827 for RM 433 with a mean of 0.658 (Table 1). The PIC value mean across 8 rice genotypes was 0.658, suggesting that the markers were highly informative (*PIC* > 0.5).

### 2.3. Parental Performance

The performance of heterotic effects can be evaluated in three ways: relative to both better parent (BP) values and mid-parent (MP) values (relative heterosis), or commercial check for yield and its component traits. These effects were examined in all studied attributes, but the level of heterosis indicated a difference from trait to trait and from the cross to cross. Significant and highly significant values of heterosis would be of interest in all traits under investigation from the rice breeder’s point of view; in the positive direction values for the traits, i.e., number of panicles per plant, panicle length, panicle weight, filled grains per panicle, 100-grain weight and grain yield per plant. On the contrary, the negative direction values were desirable heterosis towards the traits, i.e., plant height and sterility percentage. In the current analysis, heterosis over BP and MP was computed for eight agronomic, grain yields and its components traits. The data showed a broad range of heterotic patterns for all the attributes (Table 2 and Table 3). Heterosis for agronomic, grain yield along with its components traits is a very important consideration in modern plant breeding.

### 2.4. Heterosis over Mid-Parent (MP)

Heterosis effect evaluated with MP values for all agronomic, yield and its component studied traits (Table 2 and Table 3). Concerning plant height, 16 crosses out of 28 exhibited significant and highly significant heterosis over the mid-parents (HMP) and 14 of them gave undesirable positive values. The other two crosses were found to be highly significant in the negative direction of heterotic values, namely Sakha 102 × Wab880sg33 (−4.30%) and Sakha 101× Wab880sg33 (−6.25%) (Table 2). Accordingly, these two crosses could be applied in the hybrid breeding program to enhance plant height. Medium or short plant height is useful to avoid plant losses due to lodging caused by heavy winds. For this reason, negative heterosis for plant height is desirable.

In the instance of the number of panicles per plant, heterosis over MP was positive and highly significant in 20 crosses. The highest estimates were detected for the crosses Sakha 102 × Sakha 104 (29.20%), GZ7576-10-3-2-1 × Giza 179 (24.44%), Sakha 103 × IET 1444 (24.14%) and Sakha 102 × IET 1444 (23.64%) (Table 2).

Concerning panicle length, significant and positive heterotic effects compared to MP would be of concern for panicle length. There were 24 crosses that showed highly significant and positive heterotic effects as the difference from the MP, which ranged from 2.37% (Sakha 103 × Giza 179) to 21.63% (GZ7576-10-3-2-1 × IET 1444). The highest heterotic effects for panicle length were observed in GZ7576-10-3-2-1 × IET 1444, Sakha 102 × IET 1444, Sakha 103 × IET 1444, and Sakha 103 × Wab880sg33 hybrids, which gave 21.63, 17.44, 17.28 and 14.41% heterosis, respectively (Table 2).

Regarding panicle weight, 25 crosses recorded highly significant and positive estimates of heterosis over MP. The greatest estimates of heterotic effects for panicle weight were detected for crosses Sakha 102 × GZ7576-10-3-2-1 and Sakha 102 × IET 1444, which gave 13.62 and 12.03% heterosis, respectively (Table 2).

For filled grains per panicle, highly significant and positive heterotic effects were estimated for 19 cross combinations as a deviation from the MP values. The highest estimates of heterotic effects for filled grains per panicle were detected for crosses IET 1444 × Sakha 101 (30.56%), IET 1444 × Sakha 104 (26.41%) and Giza 179 × IET 1444 (25.22%) (Table 3).

Regarding sterility percentage, 26 crosses exhibited highly significant heterosis, and 21 of them gave undesirable positive heterotic values. The other 5 crosses were found to be highly significant in the negative direction (favorable) of heterotic values, namely, Sakha 103 × Wab880sg33 (−10.33%), GZ7576-10-3-2-1 × Wab880sg33 (−4.43%), GZ7576-10-3-2-1 × Sakha 101 (−3.01%), Sakha 103 × Giza 179 (−2.34%) and Sakha 103 × Wab880sg33 (−1.20%) (Table 3).

In the case of 100-grain weight, data showed highly significant and positive estimates of heterosis over MP for 24 cross mixtures. The best hybrids were Giza 179 × IET 1444 (9.67%) and Sakha 103 × Giza 179 (8.59%) (Table 3).

Concerning grain yield per plant, 21 hybrid combinations were found to exhibit significant and highly significant positive heterosis over MP, which ranged between 1.21% (Sakha 102 × Wab880sg33) and 29.32% (Sakha 103 × Sakha 104). The best crosses were Sakha 103 × Sakha 104, GZ7576-10-3-2-1 × Giza 179 and GZ7576-10-3-2-1 × IET 1444, which gave 29.32, 22.57 and 19.51% heterosis, respectively (Table 3).

### 2.5. Heterosis over Better Parent (BP)

Heterosis effect was evaluated to BP values for all agronomic, yield and its component studied attributes (Table 2 and Table 3). For plant height, 11 crosses exhibited significant HBP in the negative direction of heterotic values. The highest estimates of heterotic effects for plant height were detected for crosses Sakha 102 × Wab880sg33 (−9.86%), Sakha 101× Wab880sg33 (−8.05%) and Giza 179 × IET 1444 (−6.69%) (Table 2). Accordingly, these hybrids could be applied in the hybrid breeding program to enhance plant height.

In the instance of the number of panicles per plant, heterosis over the BP was significant and highly significant in the positive direction in 14 crosses. The highest estimated heterotic effects for the number of panicles per plant were detected for the crosses, Sakha 103 × IET 1444 (16.13%), Sakha 103 × Sakha 101 and GZ7576-10-3-2-1 × Sakha 103 (15.79%) and Sakha 102 × Sakha 104 (12.31%) (Table 2).

Concerning panicle length, significant positive heterotic effects compared to BP would be of interest for panicle length. There were 22 cross combinations that proved to be highly significant and had significant positive heterotic effects as the deviation from the BP ranged from 1.86% (Sakha 101 × Sakha 103) to 12.64% (GZ7576-10-3-2-1 × IET 1444). The best heterotic effects for panicle length were detected for the cross combinations GZ7576-10-3-2-1 × IET 1444, Sakha 102 × IET 1444, Giza 179 × Sakha 104, which gave 12.64, 11.66 and 8.84% heterosis, respectively (Table 2).

Regarding panicle weight, 16 crosses recorded highly significant and significant positive assessments of heterosis over BP. The uppermost estimates of heterotic effects for panicle weight were detected for crosses Sakha 104 × Sakha 103, Giza 179 × IET 1444, IET 1444 × Sakha 101, and IET 1444 × Wab880sg33, which gave 7.94, 7.86, 7.77 and 7.64% heterosis, respectively (Table 2).

For filled grains per panicle, significant and highly significant and positive heterotic effects were estimated for 16 cross combinations as a deviation from the BP values. The highest estimated heterotic effects for filled grains per panicle were detected for crosses, IET 1444 × Sakha 101 (27.18%), IET 1444 × Sakha 104 (19.65%), Giza 179 × IET 1444 (14.48%) and Giza 179 × Wab880sg33 (10.19%) (Table 3).

Regarding sterility percentage, 28 crosses out of the exhibited results were significant and highly significant; 16 of them gave undesirable positive heterotic values. The other 12 crosses were found to be highly significant in the negative direction of heterotic values, Sakha 103 × Wab880sg33, GZ7576-10-3-2-1 × Wab880sg33, Sakha 103 × Sakha 104 and Sakha 103 × Sakha 101, which gave −30.05, −22.01, −21.95 and −17.02% heterosis, respectively (Table 3).

In the case of 100-grain weight, data revealed significant and highly significant and positive assessments of heterosis across BP for 12 cross mixtures. The best crosses were Sakha 103 × Giza 179 (8.35%), Giza 179 × IET 1444 (7.16%), Sakha 104 × Wab880sg33 (3.87%) and GZ7576-10-3-2-1 × Wab880sg33 (3.74%) (Table 3).

Concerning grain yield per plant, 10 hybrid combinations were found to exhibit highly significant and positive heterosis over BP, which ranged from 1.37 to 11.09%. The best crosses were Sakha 103 × Sakha 104, GZ7576-10-3-2-1 × Sakha 104, Giza 179 × IET 1444 and Giza 179 × Wab880sg33, which gave 11.09, 9.62, 9.47 and 8.51% heterosis, respectively (Table 3).

### 2.6. Hybrid Prediction Based on Microsatellite Genetic Distance

GD was determined using Nei’s similarity coefficient. The relationships between genetic distance (GD)-based microsatellite marker-based with MP and BP heterosis effects for all attributes are shown in Table 4. The associations of microsatellite marker-based genetic distances with MP heterosis (MPH) were positively and significantly associated with sterility percentage (*r* = 0.390 *, *p <* 0.05) under drought stress. These results indicate that the molecular markers might be valuable for predicting the high sterility percentage. However, BP heterosis was positively and significantly associated with sterility percentage (*r* = 0.352 *, *p <* 0.05) and grain yield per plant (*r* = 0.345 *, *p <* 0.05) under drought stress. These associations indicate that high grain yield and low sterility of rice crosses can be expected from microsatellite marker-defined distances of the parents, even though the association values were not very high.

## 3. Discussion

A varied range of allelic variations was detected for each microsatellite locus. A comparable pattern of allelic difference was also identified at other loci. A different number of alleles has been found in rice utilizing microsatellite markers. Brondani et al. [19] stated a mean of 5.2 in 30 elite upland rice genotypes. Giarrocco et al. [20] detected a mean of 8.4 alleles per marker from 26 microsatellite markers in 69 accessions from Argentine. Cheng et al. [21] detected a mean number of 11.5 alleles in rice accession from 40 various origins in Africa, Asia, Europe, South America and Oceania. Cui et al. [22] identified an average of 12.5 alleles in 122 south Asia collections. Ghaley et al. [23] reported an average of 7.7 alleles per marker from 27 microsatellite markers in 352 Bhutan landraces. Das et al. [24] found an average of 7.9 alleles per marker from 23 rice microsatellites in 91 rice accessions. The value obtained here was lower than most earlier results, but it was comparable with results obtained by Meti et al. [25], who found 2.08 alleles per locus in rice.

Nevertheless, these results confirmed the conclusion of Salem and El-Zanaty [26] that the small number of markers is adequate to differentiate strongly related genotypes and carry out phylogenetic studies; consequently, selection of genotypes for the highest genetic diversity might be possible. Rice microsatellite markers revealed a mean of Polymorphic information content (PIC) value 0.658, which proves rice microsatellite markers are greatly informative. Botstein et al. [27] investigated that PIC value > 0.5 is counted as being a highly informative marker while 0.5 > PIC > 0.25 is just an informative marker, while PIC ≤ 0.25 is a slightly informative marker. Gene diversity achieved in the current study was equivalent to former findings on the genetic diversity of rice using a microsatellite study. Giarrocco et al. [20] found an average PIC value of 0.69 in 68 accessions from Argentina. Ghaley et al. [23] reported an average PIC value of 0.61 in 352 Bhutan landraces. Das et al. [24] described an average PIC value of 0.746 in 91 rice accessions. This shows that the markers were highly informative.

Predicting heterosis is practiced since heterogeneity is a dynamic phenomenon. In this research, DNA marker-dependent diversity analyses are utilized to predict heterogeneity by associating the coefficient of molecular polymorphism based on hybrid microsatellite markers with the combination of parental genotype and hybrid ability, high average morphological trait values, heterogeneity analysis on the combination of parental genotype and hybrid ability. For a higher yield/heterosis ratio, some workers stressed differential parents as heterosis is calculated in terms of rice yield. Based on several parameters, various genotypes are chosen, including pedigree, morphological, physiological, biological and DNA markers (SSRs, SNPs or ESTs) [28].

In the current research, a total of eight morphological traits were utilized to analyze the diversity of eight rice genotypes and were grouped into four groups. It has been described that genotype within a group with a high degree of variation will generate more appropriate breeding material to maximize genetic progression concerning the crop itself, provided there is a suitable complement [29]. The effectiveness of plant breeding has improved dramatically due to shortcomings in the study of diversity based on morphological data and developments in molecular techniques [30]. Microsatellites are the most accurate, functional and highly effective molecular markers for detecting polymorphisms [31]. Heterosis, however, is calculated primarily by the yield of rice. Therefore, for predicting heterosis, the use of intragenic markers is more effective. In this analysis, 11 microsatellite pairs were polymorphic.

This suggests that the study of molecular diversity is more accurate compared to the analysis of morphological diversity. Microsatellites are unaffected by the environment and DNA transcription regions, and have a higher transferability rate across species and regions that are more preserved in productivity-related genes. However, a heterosis analysis showed a maximum heterosis level in Sakha 103 × Sakha 104 and GZ7576-10-3-2-1 × Giza 179 hybrids, which gave 29.32 and 22.57% heterosis, respectively. This is because heterosis is a complex phenomenon and other factors may involve a limited range of genetic variation in the genomic regions examined among the selected parents with microsatellite markers, use of fewer markers, poor correlation with heterogeneity between marker and genes/QTLs, and interaction between genes/QTLs and error in the choice of experimental characteristics, materials and experimentation [28].

In this analysis, the highest heterotic effects for grain yield per plant, the typical heterosis of all 28 F_1_ hybrids ranged from 22.57% (GZ7576-10-3-2-1 × Giza 179) to 29.32% (Sakha 103 × Sakha 104). Positive values for parental combinations and substantial sterility percentage (*r* = 0.352 *, *p <* 0.05) and grain yield per plant (*r* = 0.345 *, *p <* 0.05) were shown in the correlation analysis between microsatellite marker-based genetic distances of parents with better-parent heterosis for grain yield and its components traits. Microsatellite-based genetic distance failed to be a dependable predictor in this analysis, as there was no meaningful association between microsatellite-based-GD and heterosis for most studied traits.

In earlier research, this marker failed to predict hybrid efficiency and heterosis, but in certain cases, the microsatellite marker may be a better predictor [32]. The utility of specific microsatellite determinants was also verified by Joshi et al. [33], who showed a significant association with hybrid efficiency. The outcome of the current study also validated the idea of main DNA markers to predict the heterosis suggested by Cho et al. [34]. Zhang et al. [28] predicted heterosis based on productivity-associated functional genes. Several variables, including germplasm type, environmental impact, genome coverage and related marker to QTL, have been proposed in former studies to explain unstable correlation coefficients.

## 4. Materials and Methods

### 4.1. Plant Materials

This research was conducted at the research farm of the Rice Research and Training Center (RRTC), Sakha, Kafr El-Sheikh, Egypt, during two rice-growing seasons (2014 and 2015). Eight rice genotypes were utilized in this study, i.e., GZ7576-10-3-2-1, Sakha 102, Sakha 103, Giza 179, Sakha 101 and Sakha 104 as local genotypes from Agriculture Research Center (ARC), Giza, Egypt, and two exotic genotypes namely, i.e., IET1444 and WAB880-SG33, obtained from the genetic stock of Rice Department, Field Crops Research Institute, Agriculture Research Center (ARC), Egypt. Name, pedigree, origin and main characteristics of the eight parent genotypes are illustrated in (Table 5).

### 4.2. Field Experiments

Eight rice genotypes were used in this research. They were grown in the field at fifteen-day intervals to defeat the difference in heading date between them (during rice-growing season 2014). Thirty days after sowing, seedlings of each genotype were individually transplanted into the permanent field in three rows, (5 m long) and 20 × 20 cm apart among plants and rows. A half diallel cross was carried out between these eight rice parents in the first growing season (without reciprocals) Griffing method 2 [35]. The bulk emasculation method was practiced by utilizing the hot water procedure according to Jodon [36].

#### 4.2.1. Drought Test

A total of 28 hybrids were made and the F_1_ seeds were cultivated as F_1_ plants in the third week of May (during the rice-growing season of 2015), and plants were transplanted individually after 30 days and adopted a spacing of 20 × 20 cm. The experimental plots were arranged in a randomized complete block design (RCBD), with three replications; each replicate consisted of one row for each parent, and three rows for F_1_. Every row was 5 m lengthy and included 25 single plants. A field experiment was irrigated only every 12 days just flushing (water deficit) (parent and hybrid genotypes cultivated), and flash irrigation was used without standing water (two weeks from transplanting till harvest). All suggested farming procedures were applied as usual for the normal rice field.

#### 4.2.2. Phenotypic Data Collection

The data were scored on a single plant base for parents and F_1_^s^. Ten random plants except border plants were chosen for phenotyping of studied characteristics according to International Rice Research Institute (IRRI) [37]. The following characters were phenotyped for parents and hybrids, i.e., plant height (cm), number of panicles per plant, panicle length (cm), panicle weight (g), filled grains per panicle, sterility percentage, 100-grain weight (g) and grain yield per plant (g).

### 4.3. Genomic DNA Extraction

Total genomic DNA was isolated from leaf tissue for every genotype. Fresh leaves from 8-week-old plants were cut as tissue samples for DNA isolation. DNA was extracted from these genotypes according to Salem [7].

### 4.4. Microsatellite Markers

In total, eleven rice microsatellite (RM) markers were selected from the Rice Genes Database (http://gramene.org (accessed on 23 June 2014)) for genotyping based on their known chromosomal location to give a uniform coverage of the rice genome. The selected RM included one RM locus from each rice chromosome. Microsatellite name, chromosomal location, sequence, motif, annealing temperature (Tm °C) and fragment size (bp) were presented in Table 6. Every 20 μL PCR mixture contained 2 μL of 50 ng μL^−1^ genomic DNA, 0.2 μL of 0.2 mM dNTPs, 1.6 μL of 1.5 mM MgCl_2_, 2 μL of 0.250 mM primers, 4 μL of 1× Taq buffer, 0.2 μL of 1 U/μL Taq polymerase and 10 μL double distilled water. The following thermal profile was used: 3 min of pre-denaturation at 94 °C, 1 min of denaturation at 94 °C, 1 min of annealing (annealing temperature is relative to the SSR primer, see annealing temperature in Table 6), 1 min of elongation at 72 °C, 45 cycles of repeated steps from denaturation to elongation and 8 min of final elongation at 72 °C. The samples were moved to 4 °C or −20 °C after the final extension (polymerization) step if they were not going to be used right away. After electrophoresis, the amplification products were isolated on 1.5% agarose gels. The bands were detected using a UV-transilluminator and photographed using a gel documentation system [7].

### 4.5. Microsatellite Marker Analysis

As stated by Akagi et al. [38] and Temnykh et al. [39], microsatellite amplification and fragment detection for rice microsatellite markers were carried out.

### 4.6. Statistical Analysis

#### 4.6.1. Determination of Heterosis

The heterosis of a single hybrid for each feature was estimated as the rise of the F_1_ means over either MP or BP, according to Mather and Jinks [40] as follows: (1) Heterosis over the mid-parents (HMP) = (F_1_ − MP)/MP × 100 and (2) Heterobeltiosis (Heterosis over the better-parent) (HBP) = (F_1_ − BP)/BP × 100, where F_1_= mean value of the first generation, MP = Mid-parent value and BP = Better parent value. To assess the heterosis significance for the above situation, L.S.D. values were assessed according to the subsequent formula proposed by Wynne et al. [41]: $L.S.D.\;\mathrm{for}\;\mathrm{HMP}\%=t.05\sqrt[{}]{3MSE/2r}.$ However, the significance of the heterosis impacts on BP is proposed in the following equation, proposed by Wynne et al. [41]: $L.S.D.\;\mathrm{for}\;\mathrm{HBP}\%=t.05\sqrt[{}]{2MSE/r}$, where, F_1_ = average value of the first generation, MP = Mid-parent value, BP = mean value of the better parent, T = tabulated *t* value at a particular possibility level and certain DF (degrees of freedom), MSE = Mean squares of error from the analysis of variance and r = number of replications.

#### 4.6.2. Cluster Analysis

Each locus of rice microsatellite (RM) bands was manually scored and analyzed as multi-allelic data. The association between eight parental lines was reconstructed using a pairwise matrix of Nei’s DA genetic distance (GD), Nei et al. [42] based on the unweighted pair-group process for the arithmetic mean (UPGMA) cluster assessment [43].

#### 4.6.3. Correlation Coefficient

Each locus of rice microsatellite (RM) bands was manually scored and analyzed as multi-allelic data. To assess the association between GD with MPH and BPH for studied attributes and yields, the Pearson linear correlation coefficients were calculated [44]. The next statistical software was applied in the result assessment: (1) SPSS (statistical package for social sciences) version 15 [45] for LSD’s analysis and Pearson correlation coefficient and (2) UPGMA cluster analysis by NTSYS-pc version 2.1 software [46].

## 5. Conclusions

Results showed that the best cross combination for grain yield was observed in Sakha 103 × Sakha 104 and GZ7576-10-3-2-1 × Giza 179, each yielding 29.32–22.57% heterosis. As a result of the poor correlation, microsatellite-based genetic distance failed to predict hybrid results and heterosis. The bad prediction was thought to be caused by a lack of dominance impact and a comparatively high genetic gap between parental lines. This study showed that for the prediction of heterosis for yield and yield-contributing characteristics in rice, a high correlation between significant microsatellite marker-based genetic distances of parents with MP and BP heterosis can be used.

## Figures and Tables

**Table 1:** Description of 11 rice microsatellites, their chromosomal location, allele size and number of alleles.

No.	Microsatellite Markers	Allele Size		Number of Alleles	Genetic Diversity
		Min Allele	Max Allele		
1	RM 1	67	119	4	0.534
2	RM 452	192	213	4	0.654
3	RM 338	178	184	4	0.456
4	RM 124	257	289	3	0.789
5	RM 162	191	244	2	0.549
6	RM118	149	165	3	0.687
7	RM 433	216	248	3	0.827
8	RM 316	194	216	4	0.597
9	RM 271	80	120	4	0.751
10	RM 144	216	295	4	0.652
11	RM 19	192	250	4	0.745
Total	-------	-------	-------	39	-------
Mean	-------	-------	-------	3.54	0.658

**Table 2:** Mid-parent heterosis (MPH) and better-parent heterosis (BPH) for 28 F_1_ hybrids from half-diallel analysis of agronomic traits under drought stress.

Hybrids	Plant Height		Number of Panicles per Plant		Panicle Length		Panicle Weight	
	MP	BP	MP	BP	MP	BP	MP	BP
P1 × P2	5.68 **	−1.76 ns	6.67 **	−1.75 ns	5.06 **	2.19 **	6.03 **	−3.13 **
P1 × P3	−2.41 ns	−4.33 *	18.92 **	15.79 **	6.91 **	5.78 **	−5.72 **	−8.39 **
P1 × P4	6.20 **	0.74 ns	24.44 **	7.69 **	13.82 **	7.14 **	2.13 **	0.10 ns
P1 × P5	7.92 **	−2.01 ns	15.97 **	11.29 **	21.63 **	12.64 **	−1.68 **	−2.22 **
P1 × P6	0.20 ns	−3.07 *	5.26 **	5.26 **	6.70 **	6.10 **	−13.24 **	−16.48 **
P1 × P7	5.16 **	−1.43 ns	−3.28 **	−9.23 **	8.94 **	4.42 **	2.34 **	−0.51 **
P1 × P8	17.58 **	15.94 **	20.93 **	8.33 **	12.81 **	1.86 **	6.85 **	6.53 **
P2 × P3	11.90 **	5.99 **	−17.65 **	−22.22 **	10.31 **	6.19 **	7.82 **	1.18 **
P2 × P4	−1.08 ns	−3.17 *	19.05 **	−3.85 **	6.60 **	3.06 **	8.33 **	−2.82 **
P2 × P5	6.69 **	4.01 **	23.64 **	9.68 **	17.44 **	11.66 **	12.03 **	2.86 **
P2 × P6	−1.65 ns	−5.63 **	20.00 **	10.53 **	4.28 **	2.00 **	5.94 **	0.33 **
P2 × P7	−1.24 ns	−2.11 ns	29.20 **	12.31 **	8.16 **	6.54 **	8.56 **	1.82 **
P2 × P8	−4.30 **	−9.86 **	3.33 **	−13.89 **	12.32 **	4.04 **	13.62 **	3.52 **
P3 × P4	1.14 ns	−2.21 ns	13.64 **	−3.85 **	2.37 **	−4.59 **	4.33 **	−0.58 **
P3 × P5	1.99 ns	−5.69 **	24.14 **	16.13 **	17.28 **	7.55 **	1.52 **	−0.82 **
P3 × P6	2.14 ns	0.77 ns	18.92 **	15.79 **	3.97 **	2.29 **	1.95 **	0.96 **
P3 × P7	3.19 *	−1.43 ns	0.84 ns	−7.69 **	−4.10 **	−9.01 **	3.91 **	3.85 **
P3 × P8	3.76 **	3.15 *	0.00 ns	−12.50 **	14.41 **	2.33 **	11.41 **	7.94 **
P4 × P5	−2.28 ns	−6.69 **	15.71 **	3.85 **	7.60 **	5.75 **	10.66 **	7.86 **
P4 × P6	6.57 **	4.41 **	−0.74 ns	−14.10 **	10.51 **	4.59 **	6.02 **	0.10 ns
P4 × P7	2.00 ns	0.72 ns	21.68 **	11.54 **	10.92 **	8.84 **	7.43 **	2.43 **
P4 × P8	−0.96 ns	−4.78 **	8.00 **	3.85 **	9.58 **	4.81 **	6.07 **	4.27 **
P5 × P6	10.36 **	3.34 *	15.97 **	11.29 **	4.76 **	−2.46 **	11.36 **	7.77 **
P5 × P7	14.19 **	10.37 **	−3.94 **	−6.15 **	10.98 **	7.06 **	0.73 **	−1.53 **
P5 × P8	5.45 **	−3.01 *	1.49ns	−5.56 **	6.30 **	3.42 **	8.57 **	7.64 **
P6 × P7	−1.11	−4.30 **	3.28 **	−3.08 **	−8.52 **	−11.84 **	5.46 **	4.39 **
P6 × P8	−6.25 **	−8.05 **	6.98 **	−4.17 **	−5.73 **	−14.44 **	1.36 **	−2.71 **
P7 × P8	9.06 **	3.58 *	−3.65 **	−8.33 **	0.33 ns	−5.75 **	3.58 **	0.40 **
L.S.D. at 0.05	2.45	2.83	1.67	1.93	0.68	0.78	0.11	0.12
L.S.D. at 0.01	3.25	3.76	2.21	2.55	0.89	1.04	0.14	0.16

*, ** significant correlations at *p ≤* 0.05 and *p ≤* 0.01 levels of probability, respectively, and ns not significant.

**Table 3:** Mid-parent heterosis (MPH) and better-parent heterosis (BPH) for 28 F_1_ hybrids from half-diallel analysis of grain yield and its components’ traits under drought stress.

Hybrids	Filled Grains per Panicle		Sterility Percentage		100-Grain Weight		Grain Yield per Plant	
	MP	BP	MP	BP	MP	BP	MP	BP
P1 × P2	6.23 **	4.07 *	9.45 **	1.27 **	−0.90 **	−5.97 **	−4.24 **	−7.05 **
P1 × P3	2.01 ns	−4.07 *	1.40 **	−4.36 **	−4.62 **	−7.67 **	8.97 **	−0.04 ns
P1 × P4	13.25 **	8.85 **	1.18 **	−9.92 **	−2.65 **	−5.97 **	22.57 **	5.78 **
P1 × P5	13.63 **	7.85 **	16.66 **	7.98 **	−3.76 **	−9.09 **	19.51 **	7.77 **
P1 × P6	−2.98 ns	−10.17 **	−3.01 **	−14.31 **	3.83 **	3.68 **	7.04 **	2.50 **
P1 × P7	2.61 ns	2.31 ns	−0.40 ns	−16.59 **	4.34 **	2.90 **	17.85 **	9.62 **
P1 × P8	9.32 **	6.32 **	−4.43 **	−22.01 **	6.95 **	3.74 **	12.87 **	1.37 *
P2 × P3	9.00 **	4.55 *	0.001 ns	−12.31 **	1.01 **	−1.06 **	−9.53 **	−14.66 **
P2 × P4	11.81 **	5.36 **	1.29 **	−2.88 **	4.04 **	2.13 **	7.25 **	−9.72 **
P2 × P5	1.72 ns	−1.52 ns	30.02 **	29.95 **	2.07 **	1.58 **	2.16 **	−10.28 **
P2 × P6	−3.05 ns	−8.48 **	14.65 **	9.02 **	0.15 **	−5.10 **	0.45 ns	−6.50 **
P2 × P7	8.58 **	6.07 **	76.37 **	58.18 **	2.06 **	−4.42 **	1.81 **	−7.87 **
P2 × P8	2.88 ns	−1.92 ns	55.89 **	35.87 **	4.42 **	−3.74 **	1.21 *	−11.46 **
P3 × P4	13.61 **	2.95 ns	58.26 **	33.86 **	8.59 **	8.35 **	−8.35 **	−26.40 **
P3 × P5	9.80 **	8.74 **	66.74 **	46.27 **	0.54 **	−1.97 **	−8.24 **	−23.35 **
P3 × P6	5.70 **	3.96 *	−1.20 **	−17.02 **	5.35 **	1.84 **	−3.64 **	−15.01 **
P3 × P7	10.32 **	3.47 ns	−2.34 **	−21.95 **	6.15 **	1.38 **	29.32 **	11.09 **
P3 × P8	18.74 **	8.79 **	−10.33 **	−30.05 **	3.84 **	−2.40 **	3.80 **	−13.61 **
P4 × P5	25.22 **	14.48 **	45.04 **	39.00 **	9.67 **	7.16 **	15.02 **	9.47 **
P4 × P6	16.52 **	4.02 *	36.01 **	34.83 **	0.15 **	−3.40 **	14.42 **	2.59 **
P4 × P7	2.09 ns	−1.61 ns	27.56 **	18.95 **	0.87 **	−3.87 **	15.74 **	6.71 **
P4 × P8	11.53 **	10.19 **	16.96 **	5.84 **	4.77 **	−1.74 **	13.52 **	8.51 **
P5 × P6	30.56 **	27.18 **	21.26 **	15.26 **	3.15 **	−2.69 **	0.12 ns	−5.99 **
P5 × P7	26.41 **	19.65 **	45.18 **	30.15 **	6.67 **	−0.55 **	10.56 **	6.92 **
P5 × P8	12.93 **	4.40 *	10.35 **	−3.86 **	6.62 **	−2.14 **	6.56 **	6.07 **
P6 × P7	10.80 **	2.31 ns	20.41 **	13.20 **	4.34 **	3.04 **	2.78 **	−0.30 ns
P6 × P8	2.28 ns	−7.69 **	5.79 **	−3.52 **	5.70 **	2.67 **	2.86 **	−3.82 **
P7 × P8	−11.83 **	−14.01 **	20.24 **	16.41 **	5.63 **	3.87 **	6.32 **	2.37 **
L.S.D. at 0.05	3.34	3.86	0.63	0.73	0.03	0.04	1.05	1.21
L.S.D. at 0.01	4.43	5.12	0.84	0.97	0.04	0.05	1.39	1.60

*, ** significant correlations at *p* ≤ 0.05 and *p* ≤ 0.01 levels of probability, respectively, and ns not significant.

**Table 4:** Correlation coefficients between microsatellite genetic similarity and mid-parent (MPH) and better parent (BPH) heterosis values for agronomic traits, yields and its components traits in diallel cross among eight rice genotypes under drought stress.

Trait	MPH		BPH	
	*r*		*r*	
Plant height	−0.016	ns	−0.025	ns
Number of panicles per plant	0.310	ns	−0.066	ns
Panicle length	−0.030	ns	0.109	ns
Panicle weight	−0.088	ns	0.166	ns
Filled grains per panicle	0.006	ns	0.126	ns
Sterility	0.390	*	0.352	*
100-grain weight	0.241	ns	0.315	ns
Grain yield per plant	−0.040	ns	0.345	*

* Significant correlations at *p ≤* 0.05 levels of probability, ns not significant.

**Table 5:** Name, pedigree, origin, group type and main characteristics of the eight rice parent genotypes.

Genotypes	Pedigree	Source of Seeds	Origin	Main Characteristics
Sakha 101 (P1)	Giza 176/Milyang 79	ARC	Egypt	Japonica type, medium grain, moderately maturing, semi-dwarf, susceptible to blast, high yielding and released in 1997
Sakha 102 (P2)	Giza177\GZ4096-7-1	ARC	Egypt	Japonica type, early maturing variety, short-grain length and susceptible to drought conditions, slightly tall stature, resistance to blast, high yielding and released in 1997
Sakha 104 (P3)	GZ 4096-8-1/ GZ4100-9-1)	ARC	Egypt	Short Japonica, Moderate tolerance to drought
GZ7576-10-3-2-1 (P4)	IR 1615-31/BG94-2349	ARC	Egypt	Indica-Japonica type, medium grain, early maturing, salt-tolerant, short stature, resistant to blast, moderately high yielding, the promising line under salinity soil conditions.
IET1444 (P5)	TN1\CO 29	ARC	India	Indica type, early maturing, semi-dwarf, resistance to blast, median grain length, tolerant to drought conditions and high yielding
WAB880-SG33 (P6)	CG 20/Wab 181-18	ARC	India	Long Indica type, drought-tolerant
Giza 179 (P7)	GZ1368 S-5/GZ626269	ARC	Egypt	Indica-Japonica type, medium grain, early maturing, salt-tolerant, short stature, resistant to blast, moderately high yielding, promising line under salinity soil condition.
Sakha 103 (P8)	Giza 177/Suweon 349	ARC	Egypt	Japonica type, early maturing, semi-dwarf, resistance to blast, short-grain length, and high yielding

ARC: Agriculture Research Center.

**Table 6:** Characteristics of 11 rice microsatellite (RM) markers, their chromosomal location, motif, annealing temperature, repeated category and fragment size.

No.	Microsatellite Markers	Chromosomal Location	Position cM	Motif	Annealing Temperature Tm (°C)	Repeat Category	Expected Fragment Size (bp)
1	RM 1	1	94.9	(GA)14	55	di	84
2	RM 19	3	94.9	(GA)17	55	di	213
3	RM 18	6	58.4	(CT)8	60	di	224
4	RM 124	2	216.4	(GA)21	60	di	106
5	RM 144	5	0	(AG)20	60	di	116
6	RM 162	10	150.1	(GATG)5	55	tetra	328
7	RM 271	9	26.7	(CT)16	55	di	126
8	RM 316	12	108.3	(GA)11	55	di	108
9	RM 388	4	0	(AT)14(GT)21	55	complex	104
10	RM 433	8	0	(CT)13	55	di	109
11	RM 452	5	26.7	(AG)11	50	di	65

RM: Rice microsatellite.

## Data Availability

All data generated or analyzed during this study already exist in this published article.
